# Cardiac MRI Findings in Patients Clinically Referred for Evaluation of Post-Acute Sequelae of SARS-CoV-2 Infection

**DOI:** 10.3390/diagnostics13132172

**Published:** 2023-06-26

**Authors:** Moritz C. Halfmann, Julian A. Luetkens, Isabel L. Langenbach, Dmitrij Kravchenko, Philip Wenzel, Tilman Emrich, Alexander Isaak

**Affiliations:** 1 Department of Diagnostic and Interventional Radiology, University Medical Center of the Johannes Gutenberg-University Mainz, 55116 Mainz, Germany; 2 German Center for Cardiovascular Research (DZHK), Partner Site Rhine-Main, 55116 Mainz, Germany; 3 Researchers for the Future (Forschende für die Zukunft), German Society of Radiology (DRG), 10587 Berlin, Germany; 4 Department of Radiology, University Hospital Bonn, 53127 Bonn, Germany; 5 Department of Radiology, Faculty of Medicine and University Hospital Cologne, University of Cologne, 50937 Cologne, Germany; 6 Department of Cardiology, University Medical Center Mainz-Center of Cardiology, Johannes Gutenberg University, 55116 Mainz, Germany

**Keywords:** long-COVID, post-acute sequelae of SARS-CoV-2 infection, PASC, cardiac magnetic resonance

## Abstract

Persistent or recurrent cardiovascular symptoms have been identified as one of the hallmarks of long-COVID or post-acute sequelae of SARS-CoV-2 infection (PASC). The purpose of this study was to determine the prevalence and extent of cardiac abnormalities in patients referred for cardiac MRI due to clinical evidence of PASC. To investigate this, two tertiary care hospitals identified all patients who were referred for cardiac MRI under the suspicion of PASC in a 2-year period and retrospectively included them in this study. Patients with previously known cardiac diseases were excluded. This resulted in a total cohort of 129 patients (63, 51% female; age 41 ± 16 years). The majority of patients (57%) showed normal cardiac results. No patient had active myocarditis or an acute myocardial infarction. However, 30% of patients had evidence of non-ischemic myocardial fibrosis, which exceeds the prevalence in the normal adult population and suggests that a possible history of myocarditis might explain persistent symptoms in the PASC setting.

## 1. Introduction

The COVID-19 pandemic caused by the Severe Acute Respiratory Syndrome Coronavirus-2 (SARS-CoV-2) has affected millions of individuals worldwide. Although COVID-19 is primarily a respiratory illness, it has become evident that it can also lead to cardiovascular complications. While the peak of the pandemic seems to have subsided, recent studies have highlighted potential long-term cardiovascular complications following the acute COVID-19 illness.

In fact, persistent or recurrent cardiovascular symptoms after recovery from the acute phase of COVID-19 have been identified as one of the hallmarks of post-acute sequelae of SARS-CoV-2 infection (PASC) [1]. Typical cardiovascular symptoms include fatigue, exertion dyspnea, exercise intolerance, tachycardia/palpitations, and chest pain [2]. While the underlying pathophysiology for these symptoms is not entirely understood and is the subject of ongoing research, it is believed to be multifactorial, including direct viral damage and immune dysregulation, potentially leading to persistent inflammation [3].

Current reports on the prevalence of cardiovascular PASC vary widely [1,3]. Possible explanations for this include inconsistent inclusion criteria and varying definitions of acute myocardial inflammation [1,3,4]. In an effort to harmonize inclusion criteria for studies on this topic, a recent consensus statement by the American College of Cardiology concluded that unselective screening of low-risk individuals is not warranted, but in the presence of persistent cardiorespiratory symptoms, cardiac MRI should be clinically considered [4]. To further specify this, the Society for Cardiovascular Magnetic Resonance has proposed optimized cardiac MRI protocols for scanning patients with active or convalescent phase COVID-19 infection and stresses that the evaluation of imaging should be strictly based on established and validated criteria such as the 2018 Lake-Louise criteria [5].

The scope of this two-center retrospective study was therefore to determine the presence and extent of cardiac abnormalities in patients with signs of PASC and clinical referrals for cardiac MRI using the previously proposed protocols and evaluation techniques. 

## 2. Materials and Methods

### 2.1. Study Population

The institutional ethics commissions of both participating centers approved this retrospective study with a waiver for informed consent. Both participating tertiary care hospitals retrospectively identified all patients who were referred for cardiac MRI within a 2-year period (May 2020 to May 2022) due to clinical signs of PASC according to current recommendations (fatigue, exertional dyspnea, exercise intolerance, tachycardia/palpitations, and chest pain > 4 weeks after infection [1,2]). Patients with previously known cardiac disease were excluded from the study.

### 2.2. Cardiac MRI

Institutional imaging protocols were in accordance with recommendations for scanning COVID-19 patients in the convalescent phase [5]. They included a short-axis cine stack covering the entire LV-extend, at least two long-axis cine views, parametric T1 and T2 mapping, as well as the administration of an intravenous contrast agent with subsequent acquisition of late gadolinium enhancement (LGE) sequences in both short- and long-axis views. 

### 2.3. Image Analysis

Cardiovascular radiologists with at least 5 years of experience (M.C.H., A.I.) performed consensual image analysis using dedicated post-processing software, including assessment of myocardial edema and LGE. Left-ventricular ejection fractions and end-diastolic volume indices were assessed in a qualitative manner using cut-offs of <55% for the ejection fraction and ≥100 mL/m^2^ for the end-diastolic volume index. Pericardial and pleural effusions were measured on axial slices and reported if they exceeded a thickness of 5 mm (pericardial effusion) and 20 mm (pleural effusion), respectively. For the evaluation of parametric mapping, a global measurement approach was used, and relaxation times of >2 standard deviations from the respective local reference values were considered abnormal to accommodate mapping sensitivity due to the differing field strengths used in this bicentric design (site 1: 1.5 Tesla, site 2: 3 Tesla) [6]. The presence of LGE was judged on a qualitative basis and, if present, further specified as ischemic or non-ischemic patterns based on localization and distribution. 

### 2.4. Statistical Analysis

Continuous data are expressed as the mean ± standard deviation. Categorical variables are expressed as frequencies, with their respective proportions expressed in percentage.

## 3. Results

A total of 129 patients were included (51% female; mean age 41 ± 16 years). The median (IQR) time between cardiac MRI and a positive reverse-transcription polymerase chain reaction test result was 4 [2,3,4,5,6,7] months. In the acute phase of COVID infection, 115/129 (89%) of patients were treated at home, while 14/129 (11%) had inpatient treatment. The most common symptoms prompting cardiac MRI were exertional dyspnea (30/129, 23%) and tachycardia/palpitations (29/129, 22%). Further baseline characteristics can be found in Table 1.

**Figure 1:**
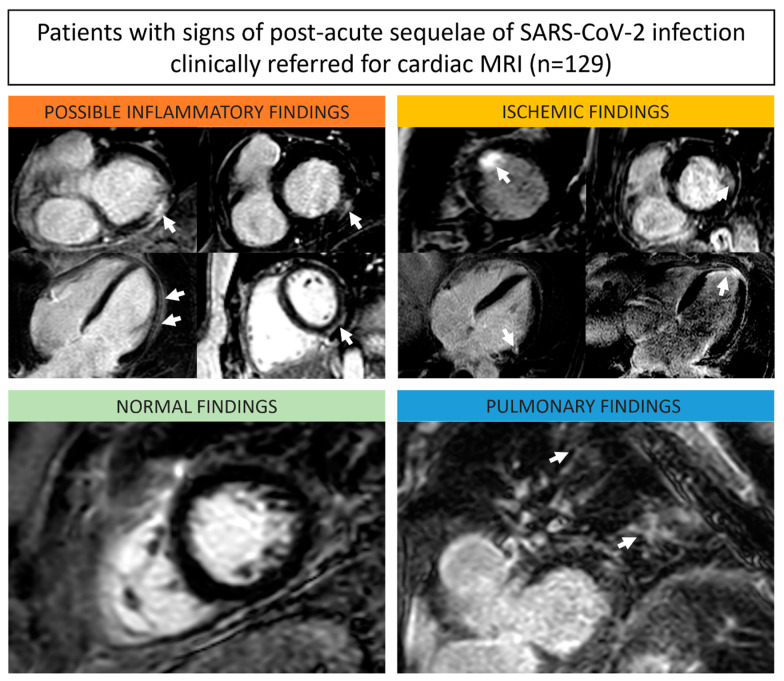
Representative late gadolinium enhancement (LGE) images of different study patients including subepicardial LGE indicating post-inflammatory fibrosis (upper left box, orange), subendocardial LGE indicating ischemic myocardial infarction (upper right box, yellow), normal myocardium (bottom left, green), and an example with diffuse enhancement of the lung, indicating pulmonary abnormalities (bottom right, blue). White arrows indicate findings of the respective type.

Volumetric analysis revealed an abnormal (<55%) ejection fraction in 27/129 (21%) and left ventricular dilation (end-diastolic volume index ≥ 100 mL/m^2^) in 25/129 (19%) of patients. A total of 18/129 (14%) patients showed pericardial effusion (>5 mm) and 6/129 (5%) had pleural effusion (>20 mm). Focal LGE was observed in 49/129 (38%) of patients, predominantly in the subepicardial layer (40/49, 82%) (Figure 1). None of the LGE lesions showed corresponding visual or quantitative myocardial edema.

Further mapping analyses revealed that 18/129 (14%) of patients had abnormal myocardial T1 relaxation times, but only 3/129 (2%) patients had abnormal T2 relaxation times. None of the patients had a simultaneous elevation of myocardial T1 and T2 times, met the 2018 Lake-Louise criteria for active myocarditis, or showed signs of acute myocardial infarction (Table 2). 

In most patients (73/129, 57%), no cardiac abnormalities were found. The most common diagnosis resulting from cardiac MRI was non-ischemic, possibly post-inflammatory fibrosis, in 39/129 (30%). Non-ischemic structural or morphological findings and ischemic cardiac findings were less common (Table 3). In 6/73 (8%) patients without cardiac abnormalities, pulmonary abnormalities were suspected (suspicion of pulmonary fibrosis/atelectasis).

## 4. Discussion

In this pre-selected cohort of patients referred for cardiac MRI with clinical suspicion of PASC, 30% of patients had signs of post-inflammatory myocardial fibrosis, which might indicate a possible history of myocarditis [4]. In 4% of patients, post-ischemic myocardial findings were found. No patient had active myocarditis or an acute myocardial infarction. Previously unknown structural cardiac disease was diagnosed in 9% of patients. In over half (57%) of the patients, cardiac results were normal; in 5% of these patients, pulmonary abnormalities were suspected to be a possible explanation for persistent symptoms. 

Previous studies have reported a wide range of prevalence for abnormal cardiac MRI findings in patients after SARS-CoV-2 infection depending on study design and inclusion criteria (26–78%, [3,7]). To improve generalizability and more closely represent a real-world scenario in imaging departments when trying to assess PASC-associated cardiovascular disease, this study focused solely on clinical referrals for cardiac MRI and strictly relied on established international guidelines for the detection of myocardial inflammation [4]. In this study, 30% of patients had non-ischemic myocardial fibrosis, which exceeds the prevalence in the normal adult population (general rates of approximately 7.9% of non-ischemic scars without direct clinical consequence have been reported in the US [8]). In a recent study, serial cardiac MRI investigations in both symptomatic and asymptomatic individuals (baseline at >4 weeks after SARS-CoV-2 infection, follow-up within a year) revealed that the presence of non-ischemic LGE at baseline was associated with the persistence or new onset of symptoms at follow-up [7]. However, in contrast to previous clinical studies, which showed rates of 8–12%, this study did not identify cases of active myocardial inflammation [3]. This is in line with results from a meta-analysis of autopsy results in which histopathological evidence of active myocarditis was rarely (<2%) found [9]. A potential explanation could be the methodological differences between previous clinical studies and the current one, namely the different time intervals between SARS-CoV-2 infection and cardiac MRI. While a longer time difference could have introduced bias through normalization of T2-relaxation times over time, it is also important to acknowledge that shorter time differences, especially those falling below the PASC cut-off of 4 weeks after infection, might represent reverberations of the initial infection [3].

However, due to the limitations of the retrospective design and the lack of baseline studies before COVID-19, there is no proof of a causal relationship between SARS-CoV-2 infection and structural, ischemic, or post-inflammatory findings. Especially for findings such as dilated cardiomyopathy, these could also be perceived as previously subclinical diseases that became overt in the PASC setting. Another limitation of this study that merits consideration is the absence of blind inter- and intra-reader variability assessments. However, since this study focused on the qualitative assessment of the cardiac abnormalities and only a few quantitative measurements were assessed, additional quantitative measurements would be of limited incremental value.

## 5. Conclusions

In this two-center cardiac MRI study, the majority of patients (57%) with clinical suspicion of cardiovascular PASC showed normal cardiac results. However, 30% of patients had evidence of non-ischemic myocardial fibrosis (exceeding the prevalence in the normal adult population), suggesting that a possible history of myocarditis might be an explanation for persistent symptoms in a PASC setting. Further previously unknown cardiac abnormalities like post-ischemic fibrosis (4%) and structural heart disease (9%) were also found as possible correlates for persistent clinical symptoms, although a causal relationship to COVID-19 seems unlikely.

## Figures and Tables

**Table 1:** Baseline characteristics and cardiac MRI indications.

General parameters	
Total cohort (n)	129 (100%)
Females	63 (51%)
Age (years)	41 ± 16
Body mass index (kg/m²)	26.6 ± 10.6
Heart rate (bpm)	71 ± 13
Indication for cardiac MRI	
Exertion dyspnea	30 (23%)
Tachycardia/palpitations	29 (22%)
Exercise intolerance	26 (20%)
Fatigue	19 (15%)
Chest pain	18 (14%)

Continuous data are expressed as the mean ± standard deviation. Categorical variables are expressed as frequencies, with their respective proportions expressed in percentage.

**Table 2:** Cardiac MRI results.

Left ventricular ejection fraction (%)	58.9 ± 9.2
≥55%	102 (79%)
<55%	27 (21%)
Left ventricular end-diastolic volume index (mL/m^2^)	85.5 ± 24.8
<100 mL/m^2^	104 (81%)
≥100 mL/m^2^	25 (19%)
Left ventricular segmental hypokinesia	1 (1%)
Late gadolinium enhancement	49 (38%)
Subepicardial	40 (82%)
Midmyocardial	3 (6%)
Subendocardial	3 (6%)
Transmural	1 (2%)
RV insertion	2 (4%)
Myocardial edema on T2-weighted imaging	0 (0%)
Abnormal T1 relaxation times	18 (14%)
Abnormal T2 relaxation times	3 (2%)
Pericardial effusion (>5 mm)	18 (14%)
Pleural effusion (>20 mm)	6 (5%)

Continuous data are expressed as the mean ± standard deviation. Categorical variables are expressed as frequencies, with their respective proportions expressed in percentage.

**Table 3:** Cardiac MRI diagnoses.

Non-ischemic cardiac findings	51 (40%)
Possible inflammatory	39 (76%)
Post-inflammation	39 (100%)
Positive for 2018 Lake Louise Criteria	0 (0%)
Structural and Morphological	12 (24%)
Valvular cardiomyopathy	6 (50%)
Dilated cardiomyopathy	3 (25%)
Hypertensive heart disease	3 (25%)
Ischemic cardiac findings	5 (4%)
No cardiac findings	73 (57%)
Pulmonary findings	6 (8%)
No extracardiac findings	67 (92%)

Categorical variables are expressed as frequencies, with their respective proportions expressed in percentage.

## Data Availability

The datasets analyzed during the current study are available from the corresponding author on reasonable request.
